# Coordinate Regulation of Antimycin and Candicidin Biosynthesis

**DOI:** 10.1128/mSphere.00305-16

**Published:** 2016-12-07

**Authors:** Thomas C. McLean, Paul A. Hoskisson, Ryan F. Seipke

**Affiliations:** a School of Molecular and Cellular Biology, Astbury Centre for Structural Molecular Biology, University of Leeds, Leeds, United Kingdom; b Strathclyde Institute of Pharmacy and Biomedical Sciences, University of Strathclyde, Glasgow, United Kingdom; UMDNJ-New Jersey Medical School

**Keywords:** natural products, regulation of secondary metabolism, secondary metabolism, *Streptomyces*

## Abstract

*Streptomyces* species produce an incredible array of high-value specialty chemicals and medicinal therapeutics. A single species typically harbors ~30 biosynthetic pathways, but only a few them are expressed in the laboratory; thus, poor understanding of how natural-product biosynthesis is regulated is a major bottleneck in drug discovery. Antimycins are a large family of anticancer compounds widely produced by *Streptomyces* species, and their regulation is atypical compared to that of most other natural products. Here we demonstrate that antimycin production by *Streptomyces albus* S4 is regulated by FscRI, a PAS-LuxR family cluster-situated regulator of the polyene antifungal agent candicidin. We report that heterologous production of antimycins by *Streptomyces coelicolor* is dependent on FscRI and show that FscRI activates the transcription of key biosynthetic genes. We also demonstrate through chromatin immunoprecipitation sequencing that FscRI regulation is direct, and we provide evidence that this regulation strategy is conserved and unique to short-form antimycin gene clusters. Our study provides direct *in vivo* evidence of the cross-regulation of disparate biosynthetic gene clusters specifying unrelated natural products and expands the paradigmatic understanding of the regulation of secondary metabolism.

**IMPORTANCE** Natural products produced by members of the phylum *Actinobacteria* underpin many industrially and medically important compounds; however, the majority of the ~30 biosynthetic pathways harbored by an average species are not expressed in the laboratory. Understanding the diversity of regulatory strategies controlling the expression of these pathways is therefore critical if their biosynthetic potential is to be explored for new drug leads. Our findings reveal that the candicidin cluster-situated regulator FscRI coordinately controls the biosynthesis of both candicidin and antimycin, which is the first observation of cross-regulation of disparate biosynthetic gene clusters specifying unrelated natural products. We anticipate that this will emerge as a major strategy by which members of the phylum *Actinobacteria* coordinately produce natural products, which will advance our understanding of how the expression of secondary metabolism is controlled and will aid the pursuit of “silent” biosynthetic pathway activation.

## Abstract (precis)

Natural products produced by members of the phylum *Actinobacteria* underpin many industrially and medically important compounds; however, the majority of the ~30 biosynthetic pathways harbored by an average species are not expressed in the laboratory. Understanding the diversity of regulatory strategies controlling the expression of these pathways is therefore critical if their biosynthetic potential is to be explored for new drug leads. Our findings reveal that the candicidin cluster-situated regulator FscRI coordinately controls the biosynthesis of both candicidin and antimycin, which is the first observation of cross-regulation of disparate biosynthetic gene clusters specifying unrelated natural products. We anticipate that this will emerge as a major strategy by which members of the phylum *Actinobacteria* coordinately produce natural products, which will advance our understanding of how the expression of secondary metabolism is controlled and will aid the pursuit of “silent” biosynthetic pathway activation.

## INTRODUCTION

Microbial natural products underpin most of the pharmaceuticals in clinical use (1), and filamentous members of the phylum *Actinobacteria*, such as *Streptomyces* species, are prolific producers of these diverse small molecules. *Streptomyces* species typically harbor between 20 and 50 biosynthetic pathways, but only a few them are expressed under common laboratory conditions (2). The biochemical diversity encoded by these silent or unproductive biosynthetic pathways is widely believed to be a tremendous untapped source of new antibacterial agents and other therapeutics. The regulation of natural-product biosynthesis is complex and typically involves pleiotropic global regulators that either directly activate or repress biosynthetic genes or do so via cluster-situated activators or repressors (3). Major roadblocks preventing the exploitation of silent biosynthetic pathways are a lack of insight into their regulation and limited technology for activating their expression. Advances in this area have significant potential to unlock the diversity of natural products for drug discovery.

Antimycin-type depsipeptides are a large class of natural products widely produced by *Streptomyces* species (see references 4 and 5 for recent reviews). Antimycins are the archetypal members of this family and have been known for more than 65 years (6). They possess a myriad of biological properties, including antifungal, insecticidal, and nematocidal activities, owing to their ability to inhibit mitochondrial cytochrome *c* reductase (7), and are used commercially as a fish pesticide (brand name, Fintrol) (8). Recently, antimycins were found to be potent and selective inhibitors of the mitochondrial Bcl-2/Bcl-X_L_-related antiapoptotic proteins that are overproduced by cancer cells and confer resistance to chemotherapeutic agents whose mode of action is activation of apoptosis (9).

**FIG 1:**
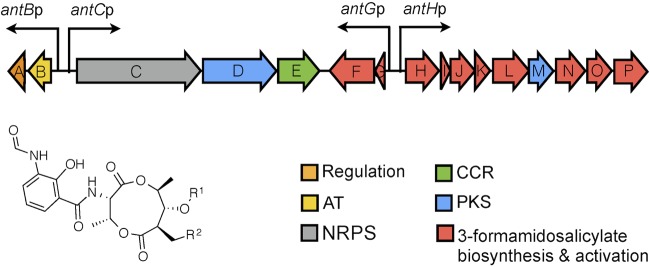
Schematic representation of the antimycin biosynthetic gene cluster of *S. albus* S4. Genes are color coded to indicate their functions. AT, acyltransferase; CCR, crotonyl-CoA carboxylase/reductase. In antimycin A_1_, R^1^ is COCH(CH_3_)CH_2_CH_3_ and R^2^ is (CH_2_)_4_CH_3_. In antimycin A_2_, R^1^ is COCH(CH_3_)_2_ and R^2^ is (CH_2_)_4_CH_3_. In antimycin A_3_, R^1^ is COCH_2_CH(CH_3_)_2_ and R^2^ is (CH_2_)_2_CH_3_. In antimycin A_4_, R^1^ is COCH(CH_3_)_2_ and R^2^ is (CH_2_)_2_CH_3_.

The hybrid nonribosomal peptide synthetase (NRPS)/polyketide synthase (PKS) pathway encoding the biosynthesis of antimycins remained enigmatic until it was elucidated recently in *Streptomyces albus* S4 (10, 11). The ~25-kb antimycin (*ant*) biosynthetic gene cluster is composed of 15 genes organized into four polycistronic transcription units, *antBA*, *antCDE*, *antGF*, and *antHIJKLMNO* (Fig. 1) (12). The *antFGHIJKLNO* genes specify the biosynthesis of the unusual starter unit 3-formamidosalicyl coenzyme A (CoA) (13–15). AntCD make up the hybrid NRPS/PKS assembly line, while AntE and AntM are crotonyl-CoA reductase and discrete ketoreductase homologs, respectively, and AntB is an acyltransferase responsible for the acyloxyl moiety and the chemical diversity observed at R^1^ (Fig. 1) (14, 16, 17).

The *ant* genes are expressed during vegetative growth and are significantly downregulated during aerial growth, such that the gene cluster is not constitutively active and suggesting that its expression is tightly regulated (12). The *ant* gene cluster harbors a single cluster-situated regulator, an extracytoplasmic function RNA polymerase sigma (σ) factor named σ^AntA^, which only activates the transcription of operons *antGF* and *antHIJKLMNO*, suggesting that the regulator(s) controlling the expression of *antBA* and *antCDE* must be encoded at another locus (12). We consistently have been unable to heterologously produce antimycins by using a variety of *Streptomyces* strains, including *S. coelicolor*, which had previously been reported to be a suitable host for the expression of this pathway (18). We presumed that this anomaly related to the unknown regulator(s) controlling the expression of *antBA* and *antCDE* and sought to identify and characterize the transcription factor(s) in this study.

Here we demonstrate that antimycin production in *S. albus* S4 is regulated by FscRI, a LuxR family cluster-situated regulator of the polyene antifungal agent candicidin. We report that heterologous production of antimycins by *S. coelicolor* is dependent on FscRI and show that FscRI activates the expression of *antBA* and *antCDE*. We also demonstrate through chromatin immunoprecipitation (ChIP) and sequencing that FscRI regulation is direct and provide evidence that this regulation strategy is conserved and unique to short-form *ant* gene clusters. Our findings reveal coordinate control of antimycin and candicidin biosynthesis, thereby providing direct *in vivo* evidence of the cross-regulation of disparate biosynthetic gene clusters specifying unrelated natural products, and expand our paradigmatic understanding of the regulation of secondary metabolism.

## RESULTS AND DISCUSSION

### Identification of FscRI binding sites associated with antimycin biosynthesis.

We previously characterized σ^AntA^ as an activator of *antGF* and *antHIJKLMNO* expression and postulated that the regulator(s) governing the expression of the remaining operons (*antBA* and *antCDE*) must be encoded elsewhere in the *S. albus* S4 genome (Fig. 1) (12). Recently, increased antimycin production was observed in a strain of *S. albus* J1074 engineered to heterologously overproduce PimM (19). PimM is a cluster-situated activator of pimaricin (or natamycin) biosynthesis and belongs to the PAS-LuxR family of transcriptional regulators, which harbor an N-terminal PER-ARNT-SIM) domain that recognizes stimuli such as light, oxygen, redox potential, or other ligands to modulate the activity of a C-terminal helix-turn-helix DNA-binding motif (20–22). Orthologs of PimM also control the production of related polyene antifungal agents, amphotericin (AmphDIV), nystatin (NysRIV), filipin (PteF), and candicidin (FscRI) and a coronafacic-acid-like phytotoxin (CfaR) (23–27). The amino acid sequences of polyene PAS-LuxR regulators are 65 to 94% identical and show functional cross complementarity, a consequence of their nonperfect inverted repeat binding sequence (5′-CTVGGGAWWTCCCBAG-3′) (28). *S. albus* S4 also produces candicidin (29) and harbors an FscRI ortholog (11); thus, we hypothesized that FscRI was the missing regulator of antimycin biosynthesis.

**FIG 2:**
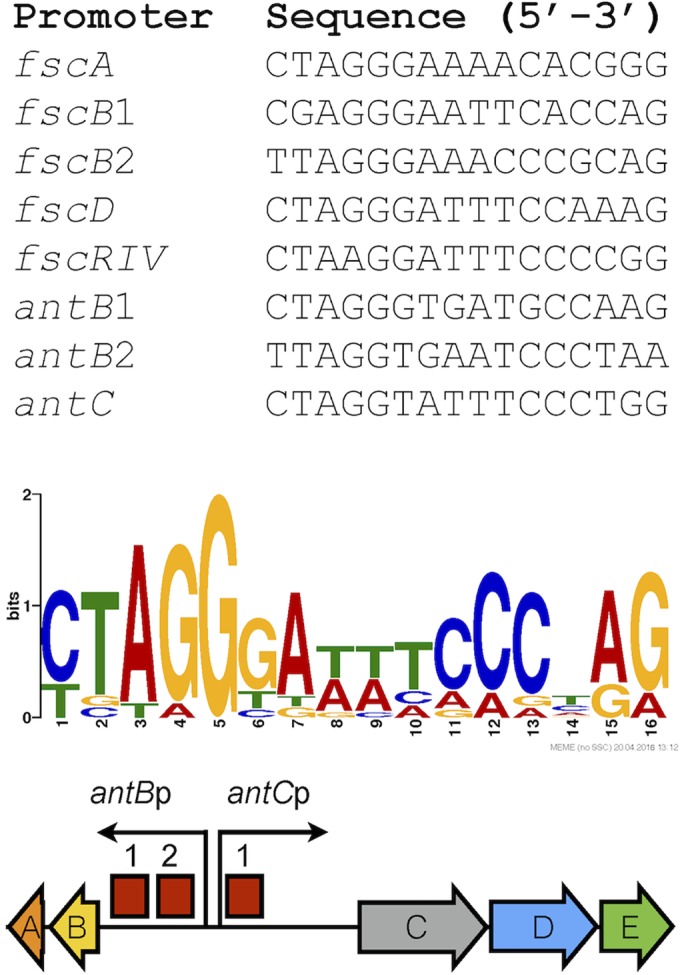
FscRI binding sites within the *S. albus* S4 antimycin gene cluster. The top panel shows experimentally verified FscRI binding sites upstream of genes within the candicidin biosynthetic gene cluster (*fscA*, *fscB1*, *fscB2*, *fscD*, and *fscMI*) and putative FscRI binding sites upstream of *antB* and *antC* within the *ant* biosynthetic gene cluster. The middle panel displays the WebLogo (61) for the verified and putative FscRI binding sites above, and the bottom panel shows the relative locations of FscRI binding sites (as red boxes) upstream of *antB* and *antC*.

FscRI was recently characterized in the candicidin producer *Streptomyces* sp. strain FR-008 and is required for the expression of 16 out of 21 genes within the gene cluster (26). DNA motifs consistent with those recognized by PimM-type regulators were identified upstream of *fscA*, *fscB*, and *fscD*, which each encode a type I PKS, and *fscRIV*, which is a LAL regulator (a large ATP-binding regulator of the LuxR type) (26). The amino acid sequences of FscRI^FR-008^ and FscRI^S4^ are 100% identical, and inspection of the *S. albus* S4 genome sequence revealed the presence of DNA motifs identical to those upstream of *fscA*, *fscB*, *fscD*, and *fscMI* in *Streptomyces* sp. strain FR-008 (Fig. 2). Thus, we used these DNA sequences with the MEME suite (30) to search for similar motifs within the antimycin gene cluster, which resulted in the identification of two putative FscRI binding sites upstream of *antBA* and one upstream of *antCDE* (Fig. 2). Taken together, these findings suggest that the cluster-situated regulator of candicidin biosynthesis, FscRI, may directly activate the expression of both *antBA* and *antCDE*.

### FscRI is required for antimycin production.

**FIG 3:**
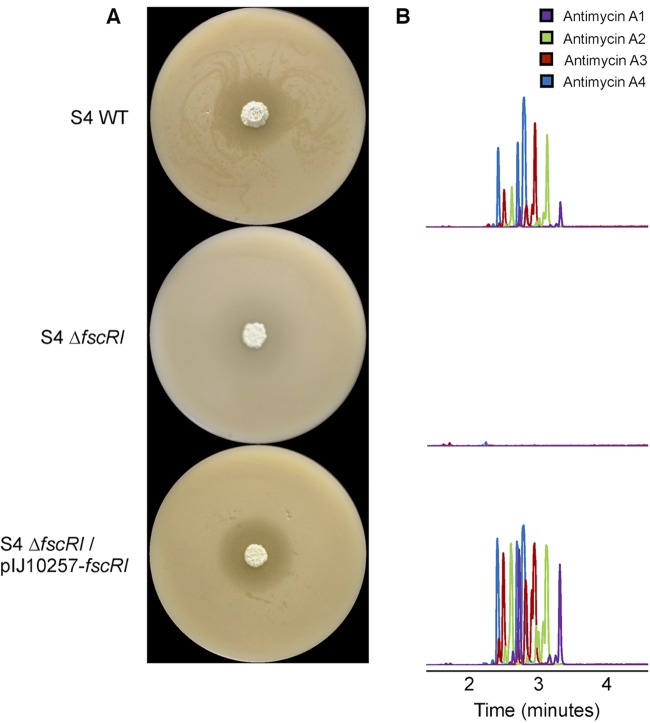
FscRI is required for the biosynthesis of antimycins by *S. albus* S4. (A) *S. albus* S4 strains challenged with *C. albicans*. The Δ*fscRI* mutant does not show detectable bioactivity against *C. albicans* compared to the wild-type (WT) and complemented (Δ*fscRI*/pIJ10257-*fscRI*) strains. (B) LC-HRESI-MS analysis of chemical extracts prepared from strains shown in panel A; the extracted ion chromatograms (M+H)^+^ for antimycins A_1_ to A_4_ are shown for each strain.

Our bioinformatic analyses led us to hypothesize that FscRI^S4^ activates the transcription of *antBA* and *antCDE* and is thus likely to be required for the production of antimycins. To investigate this possibility, we deleted the *fscRI* gene by CRISPR/Cas9 editing and tested the resulting mutant (Δ*fscRI*) against *Candida albicans* in a bioassay. As predicted, the Δ*fscRI* mutant strain no longer inhibited the growth of *C. albicans*, which is consistent with loss of antimycin and candicidin production (Fig. 3) (12). Complementation of this mutant with pIJ10257-*fscRI*, which contains the *fscRI* gene under the control of the constitutive ermE* promoter, restored bioactivity against *C. albicans* to wild-type levels and verified that loss of bioactivity was not due to other mutational events (Fig. 3). Ultrahigh-performance liquid chromatography–high-resolution electrospray ionization–mass spectrometry (LC-HRESI-MS) confirmed that compounds with molecular formulae consistent with antimycins A_1_ to A_4_ were only present in chemical extracts prepared from wild-type and Δ*fscRI* mutant *S. albus* S4 strains harboring pIJ10257-*fscRI* but not in those from the Δ*fscRI* mutant (Fig. 3). Taking these findings together, we conclude that FscRI is required for the production of antimycins and candicidin by *S. albus* S4. Given the rather flexible and conserved binding site of PAS-LuxR regulators, it is conceivable that orthologs of FscRI could also cross-regulate a gene cluster(s) other than the one in which they are encoded. This is an intriguing possibility that has not been rigorously explored.

To our knowledge, cross-regulation of disparate natural-product biosynthetic gene clusters by a cluster-situated regulator has only been demonstrated once previously. In *Streptomyces clavuligerus*, the cephamycin and clavulanic acid gene clusters comprise a contiguous “supercluster” (31). The biosynthesis of both cephamycin and clavulanic acid is coordinately controlled by CcaR, a SARP (*Streptomyces* antibiotic regulatory protein)-type activator harbored within the cephamycin gene cluster (32–34). It is interesting that not only are these gene clusters contiguous, but unlike antimycin and candicidin, both molecules are structurally similar and possess complementary biological activities (cephamycin is a β-lactam antibiotic, and clavulanic acid is a β-lactamase inhibitor).

### Heterologous production of antimycins by *S. coelicolor* requires FscRI.

**FIG 4:**
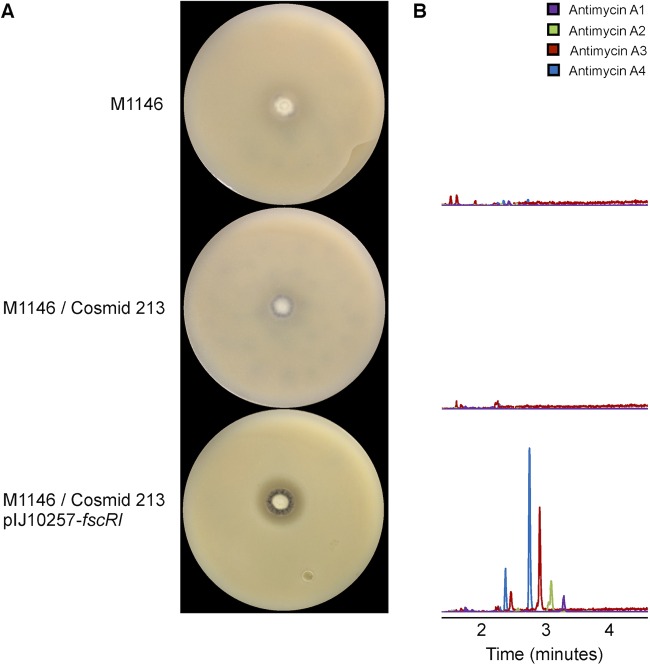
Heterologous production of antimycins by *S. coelicolor* is FscRI dependent. (A) *S. coelicolor* M1146 strains challenged with *C. albicans*. Only M1146 harboring both cosmid 213 and pIJ10257-*fscRI* inhibits the growth of *C. albicans* compared to M1146 and M1146 harboring cosmid 213. (B) LC-HRESI-MS analysis of chemical extracts prepared from strains shown in panel A; the extracted ion chromatograms (M+H)^+^ for antimycins A_1_ to A_4_ are shown for each strain.

Yan et al. cloned the *ant* gene cluster from *Streptomyces* sp. strain NRRL 2288 and heterologously produced antimycins with *S. lividans* and *S. coelicolor* M145 (18). To our surprise, even though the *ant* gene cluster nucleotide sequences of NRRL 2288 and S4 are >97% identical (18), we have consistently been unable to repeat these findings by using both gene clusters and multiple genetic backgrounds, including *Streptomyces* sp. strain S3, *S. lividans* 66, and *S. coelicolor* M145, M1146, M1152, and M1153 (29, 35, 36; R. F. Seipke and M. I. Hutchings, unpublished data). Previously, we presumed that poor availability of one of more biosynthetic precursors (i.e., tryptophan, threonine, pyruvate, and acyl-CoA) precluded the production of antimycins and/or that the pathway was simply not expressed by the heterologous hosts under our growth conditions. But given our observations described above, we hypothesized that *S. coelicolor* did not produce antimycins because of a lack of FscRI rather than as a result of culture conditions. To test this hypothesis, we introduced cosmid 213, containing the entire S4 *ant* gene cluster (10), into *S. coelicolor* M1146 (36) and also introduced pIJ10257-*fscRI*. The resulting strains were then tested for the ability to inhibit the growth of *C. albicans* by bioassay. Consistent with our hypothesis, M1146 harboring solely cosmid 213 or pIJ10257-*fscRI* did not inhibit the growth of *C. albicans*; however, the cointegrant harboring both cosmid 213 and pIJ10257-*fscRI* inhibited *C. albicans* growth (Fig. 4; see Fig. S1 in the supplemental material). We recapitulated this experiment with the *ant* gene cluster from NRRL 2288 and the pAL2602 cosmid clone generated by Yan et al. (18) and obtained identical results (see Fig. S1). The data set for the heterologous expression of the S4 *ant* gene cluster was corroborated by LC-HRESI-MS detection of antimycins A_1_ to A_4_ in chemical extracts prepared from M1146 harboring cosmid 213 and pIJ10257-*fscRI* and their absence from M1146 alone or harboring only cosmid 213 (Fig. 4). These results demonstrate that FscRI is required by *S. coelicolor* for heterologous production of antimycins with two different *ant* cluster cosmid clones. More importantly, however, our findings suggest that integral components of biosynthetic pathways may be encoded by disparate loci, which is consistent with recent observations that the *Pseudonocardia* metabolite gerumycin is encoded by two loci separated by over 90 kb (37).

10.1128/mSphere.00305-16.4 Figure S1  Bioactivity of *S. coelicolor* M1146 harboring pAL2602 is FscRI dependent. *S. coelicolor* M1146 harboring both pAL2602 and pIJ10257-*fscRI* antagonizes the growth of *C. albicans*, while M1146 harboring only pAL2602 or pIJ10257-*fscRI* does not. Download Figure S1, TIF file, 2.3 MB. Copyright © 2016 McLean et al. 2016 McLean et al. This content is distributed under the terms of the Creative Commons Attribution 4.0 International license.

### FscRI activates the expression of *antBA* and *antCDE*.

**FIG 5:**
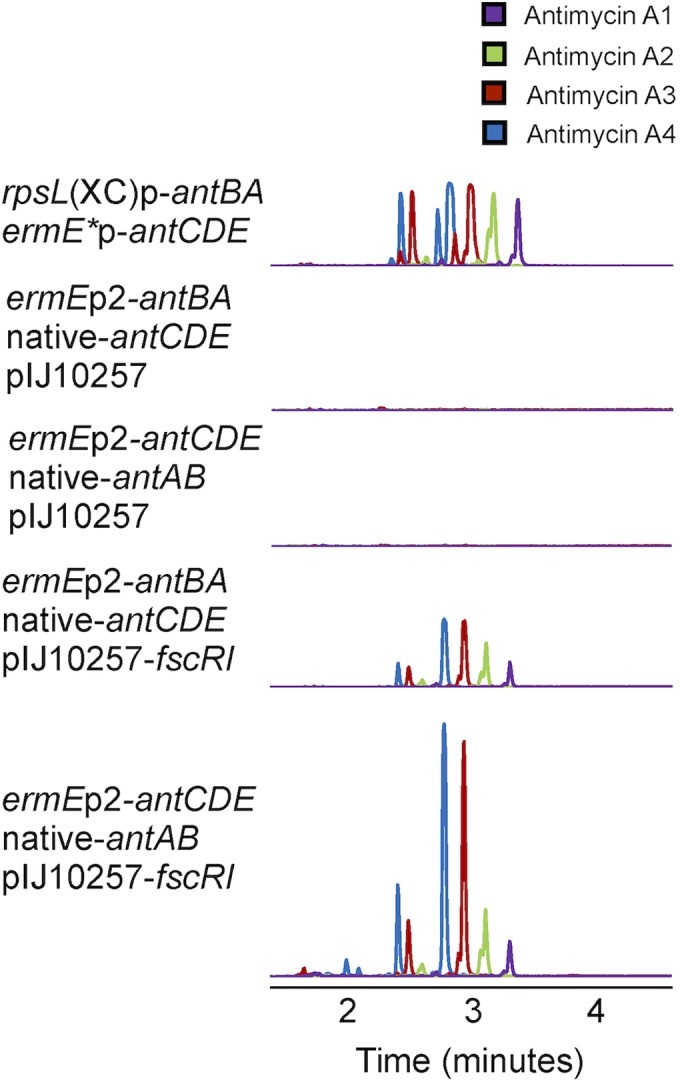
FscRI activates *antBA* and *antCDE* expression. LC-HRESI-MS analysis of chemical extracts prepared from *S. coelicolor* M1146 harboring variants of cosmid 213 engineered as shown here and pIJ10257 or pIJ10257-*fscRI* as indicated. The extracted ion chromatograms (M+H)^+^ for antimycins A_1_ to A_4_ are shown for each strain.

The observation that FscRI is required for heterologous production of antimycins by *S. coelicolor* suggests that it activates the expression of both *antBA* and *antCDE*. To evaluate this hypothesis, we first engineered cosmid 213 such that *antBA* and *antCDE* were expressed from the *rpsL*(XC) promoter (38) and the ermE* promoter (39), respectively. As we expected, M1146 harboring solely this engineered cosmid displayed an FscRI-independent ability to produce antimycins, which is consistent with bioinformatic data showing the absence of FscRI binding sites elsewhere within the *ant* gene cluster (Fig. 5). Next, we engineered two more variants of cosmid 213 such that the expression of only *antBA* or only *antCDE* was driven by *ermE*p2, leaving the native FscRI-dependent promoters of *antCDE* and *antBA* intact, respectively. The engineered cosmids and either pIJ10257 or pIJ10257-*fscRI* were mobilized to M1146, and the ability of the resulting strains to produce antimycins was assessed by LC-HRESI-MS. As anticipated, antimycins A_1_ to A_4_ were detected only in chemical extracts prepared from M1146 harboring pIJ10257-*fscRI* and cosmid 213 with either *ermE*p2-driven *antBA* or *antCDE* and not those generated from M1146 harboring just pIJ10257 and cosmid 213 with *ermE*p2-driven *antBA* or *antCDE* (Fig. 5). These data provide *in vivo* evidence that FscRI may activate the expression of both *antBA* and *antCDE* and are consistent with our hypothesis that FscRI acts on these promoters.

### FscRI directly activates the transcription of *antBA* and *antCDE*.

**FIG 6:**
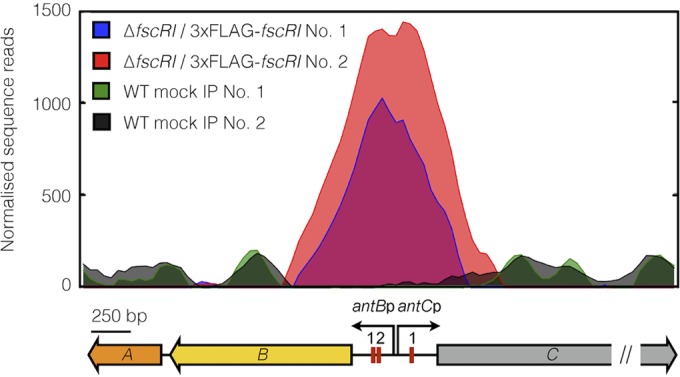
3×FLAG-FscRI binds to the *antBA* and *antCDE* promoters *in vivo*. Shown is a graphic representation of normalized sequence reads mapped to the intergenic region of *antB-antC*, which is shown at the bottom. The double slash indicates that the sequence window presented does not contain the entire *antC* coding sequence. The genomic coordinates depicted are nucleotides 16873 to 20973 of contig CADY01000091.1 of the *S. albus* S4 genome (11). WT, wild type; IP, immunoprecipitation.

The simplest interpretation of our bioinformatic analysis and heterologous expression data is that FscRI activation of *antBA* and *antCDE* is direct. We initially sought to verify this hypothesis by performing electrophoretic mobility shift assays with purified FscRI protein; however, FscRI harboring either an N-terminal or a C-terminal hexahistidine tag was insoluble when overproduced by *Escherichia coli* (data not shown), which was surprising given than His_6_-FscRI^FR-008^ was reportedly soluble (26). Nevertheless, we adopted a ChIP-sequencing approach to determine if the *antB* and *antC* promoters were bound by FscRI *in vivo*. We complemented the Δ*fscRI* mutant with an N-terminal 3×FLAG-tagged version of FscRI expressed from the ΦC31 integration site. The resulting strain (Δ*fscRI*/pSETNFLAG-*fscRI* mutant) inhibited the growth of *C. albicans* and that of the wild-type strain equally (see Fig. S2 in the supplemental material). ChIP-sequencing was carried out with anti-FLAG antibodies and lysate from the Δ*fscRI*/pSETNFLAG-*fscRI* mutant and wild-type strains cultivated in LB, which facilitates the production of both antimycin and candicidin. Immunoprecipitated DNA from two biological replicates of S4 wild-type and Δ*fscRI*/pSETNFLAG-*fscRI* and nonimmunoprecipitated chromosomal DNA were sequenced with the Illumina HiSeq3000 platform and processed as described in Materials and Methods. As we anticipated, the numbers of sequencing reads that mapped to the *antB* and *antC* promoter regions were enriched for both biological replicates of Δ*fscRI*/pSETNFLAG-*fscRI* compared to that of the wild-type mock-immunoprecipitated control (Fig. 6). These data provide definitive evidence that FscRI binds to the *antB* and *antC* promoters and likely promotes the transcription of *antBA* and *antCDE* (Fig. 6).

10.1128/mSphere.00305-16.5 Figure S2  Schematic of the pSETNFLAG-*fscRI* plasmid (left) and antifungal bioactivity of Δ*fscRI* expressing 3×FLAG-FscRI against *C. albicans*. *aac*(*3*)*IV*, apramycin resistance cassette; *oriT*, origin of transfer; *attP*, ΦC31 attachment site. The sequences of pSETNFLAG and its parent pSET152-*ermE*p are available at http://www.ryanseipkelab.com/tools.html. Download Figure S2, TIF file, 1.6 MB. Copyright © 2016 McLean et al. 2016 McLean et al. This content is distributed under the terms of the Creative Commons Attribution 4.0 International license.

10.1128/mSphere.00305-16.6 Figure S3  Clustal Ω alignment of the *antB-antC* intergenic region of S-form *ant* gene clusters. The putative start codons for *antB* (bold, red, reverse orientation) and *antC* (bold, blue, forward orientation) and the three conserved FscRI binding sites are shaded gray. Download Figure S3, TIF file, 1.3 MB. Copyright © 2016 McLean et al. 2016 McLean et al. This content is distributed under the terms of the Creative Commons Attribution 4.0 International license.

### FscRI regulation of antimycin biosynthesis is conserved for S-form antimycin gene clusters.

We and others previously identified 14 *ant* gene clusters that were classified as short-form (S-form, 15 genes), intermediate-form (I-form, 16 genes), and long-form (L-form, 17 genes) clusters (4, 18). There are six taxa, all related to *S. albus* S4, that encode S-form *ant* gene clusters: *S. albus* S4, *S. albus* J1074, *Streptomyces* sp. strain SM8, *Streptomyces* sp. strain NRRL2288, *Streptomyces* sp. strain LaPpAH-202, and *Streptomyces* sp. strain CNY228 (40). I-form *ant* gene clusters are encoded by two species, *Streptomyces* sp. strain 303MFCol5.2 and *Streptomyces* sp. strain TOR3209, which lack either *antQ* or *antP*, respectively. L-form *ant* gene clusters are encoded by six taxa, *S. ambofaciens* ATCC 23877, *S. blastmyceticus* NBRC 12747, *S. gancidicus* BKS 13-15, *S. griseoflavus* Tü4000, *S. hygroscopicus* subsp. *jinggangensis* 5008, and *S. hygroscopicus* subsp. *jinggangensis* TL01. To determine if FscRI cross-regulation of antimycin biosynthesis is likely to be widespread, we first looked for orthologs of FscRI in genomes of antimycin producers. *S. blastmyceticus* and *Streptomyces* sp. strain NRRL2288 were omitted from this analysis because their genome sequences are not available. A tBLASTn search of a local blast database for the deduced amino acid sequence of FscRI^S4^ revealed that organisms harboring an S-form *ant* gene cluster also harbor an FscRI ortholog (>99% amino acid sequence identity), whereas the top tBLASTn hits for taxa harboring either an I- or an L-form *ant* gene cluster displayed a rather low level of shared amino acid identity (36 to 46%), with the exception of one organism, *Streptomyces* sp. strain TOR3209, which possesses an ortholog of FscRI^S4^ (79% identical; see Table S3 in the supplemental material). Next, we closely inspected all 14 *ant* gene clusters for the presence of FscRI DNA-binding motifs, which revealed that only S-form *ant* gene clusters harbor a motif consistent with that identified in this study, which was somewhat surprising, as we expected *Streptomyces* sp. strain TOR3209 to also harbor this motif, given that it encodes what appears to be an FscRI ortholog (see Fig. S4 in the supplemental material). Taking these findings together, we conclude that cross-regulation of antimycin biosynthesis by FscRI is likely a conserved regulatory strategy for bacteria that harbor an S-form *ant* gene cluster but was not a strategy adopted by taxa possessing I-form or L-form variants. The regulation of I-form and L-form *ant* gene clusters has not yet been investigated, so the regulatory mechanism(s) controlling the expression of *antBA* and *antCDE* is unknown; however, bioinformatic analyses suggest that, like S-form gene clusters, the genes encoding the biosynthesis and activation of 3-formamidosalicylate (*antGF* and *antHIJKLMNO*) are regulated by σ^AntA ^ (12).

10.1128/mSphere.00305-16.7 Figure S4  Schematic of the theophylline riboswitch cassette AprTheo. P1, prime site 1; P2, prime site 2; P3, prime site 3; *aprR*, apramycin resistance; 6×His, hexahistidine affinity purification tag. The riboswitch is represented by a hairpin. FRT sites are for excision of the resistance marker by the Flp recombinase. The sequence of the plasmid harboring this cassette is available at http://www.ryanseipkelab.com/tools.html. Download Figure S4, TIF file, 0.8 MB. Copyright © 2016 McLean et al. 2016 McLean et al. This content is distributed under the terms of the Creative Commons Attribution 4.0 International license.

### Antimycin and candicidin do not act synergistically.

It is reasonable to assume that coordinate production of antimycin and candicidin may confer a competitive advantage upon the producer, akin to coordinate control of the β-lactam antibiotic cephamycin and the β-lactamase inhibitor clavulanic acid described above (31–34). One intriguing explanation for this could be that the compounds act synergistically to inhibit the growth of nearby fungi. We therefore used *C. albicans* to measure the MICs of antimycin (0.125 μg/ml) and candicidin (2 μg/ml) alone, as well as the MICs of pairwise mixtures of these agents, which allowed us to determine the fractional inhibitory concentration (FIC) index (see Materials and Methods). We calculated an FIC index of 2, which indicates that antimycin and candicidin do not interact synergistically or additively but also do not act antagonistically. This was surprising to us, because a slight synergistic effect against the fungus *Escovopsis weberi* was recently reported (41); however, an FIC index was not calculated, which limits interpretation and comparison of the data. An alternative possibility is that coordinate production of antimycin and candicidin serves to limit the development of resistance to either agent. The target of antimycin is cytochrome *c* reductase, and resistance can be conferred by a single point mutation (42), whereas development of resistance to candicidin and other polyene antifungal agents relies upon alteration of sterol biosynthesis, which incurs a significant fitness cost (43). It is also possible that coordinate production of these compounds relates to the monomeric precursors utilized by each pathway. For instance, the candicidin gene cluster harbors three genes (*pabABC*) responsible for the production of *p*-aminobenzoic acid (PABA) (44). *In vitro* studies of purified PabC revealed that its PABA synthase activity is inhibited by the aromatic amino acids tyrosine, phenylalanine, and tryptophan (45). It is conceivable that repression of PabC can be alleviated by AntFGHIJKLMNO, which utilize tryptophan to generate the 3-formamidosalicylate starter unit and may underpin the rationale for the coordinate production of antimycin and candicidin.

### Model of the regulation of antimycin biosynthesis.

**FIG 7:**
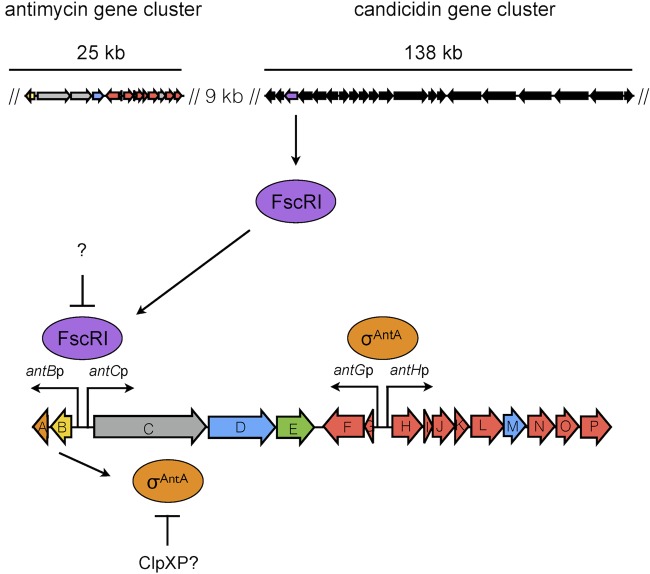
Model of regulation of antimycin biosynthesis. The top panel displays the relative locations of the antimycin and candicidin gene clusters in the *S. albus* S4 chromosome. In the bottom panel, FscRI activates the transcription of *antBA* and *antCDE*, which results in production of the core AntC/AntD NRPS/PKS megasynthase and production of the discrete acyltransferase AntB and σ^AntA^, which in turn activates the transcription of the ketoreductase gene *antM* and nine genes (*antFGHIJKLNO*) required for the biosynthesis and activation of the 3-formamidosalicylate precursor utilized by AntC. σ^AntA^ does not possess a cognate anti-σ factor and instead appears to be inactivated by the ClpXP protease.

Our model of the regulation of antimycin biosynthesis is depicted in Fig. 7. FscRI activates the expression of *antBA* and *antCDE*, which in turn results in the σ^AntA^-mediated expression of *antGF* and *antHIJKLMNO* (12). FscRI does not activate its own production; however, the expression of *fscRI* is regulated by a positive feedback loop where FscRI activates FscRIV, which in turn activates the transcription of *fscRI* (26). This observation, combined with our findings here and the fact that the *ant* gene cluster is expressed during vegetative growth and downregulated upon the onset of morphological differentiation (12), suggests that the ligand(s) recognized by the FscRI PAS domain, and perhaps all PAS domains of polyene PAS-LuxR regulators, is available only during vegetative growth. Following the inactivation of FscRI, the cell must have a strategy in place to prevent σ^AntA^ from activating its targets. Indeed, in the absence of a cognate anti-sigma factor that would ordinarily perform this task, σ^AntA ^seems to have evolved to be a direct substrate for the ClpXP protease (12).

As our understanding of the regulation of microbial natural-product biosynthesis increases, we anticipate that cross-regulation by cluster-situated regulatory proteins will emerge as a major strategy by which members of the phylum *Actinobacteria* coordinately produce selected natural products.

## MATERIALS AND METHODS

### Growth media, strains, cosmids, plasmids, and other reagents.

*E. coli* strains were propagated on Lennox agar (LA) or broth (LB) (35), and *Streptomyces* strains were cultivated with LA, LB, and mannitol-soya flour (MS) agar or broth (35) Culture medium was supplemented with antibiotics as required at the following concentrations: apramycin, 50 μg/ml; carbenicillin, 100 μg/ml; chloramphenicol, 25 μg/ml; hygromycin, 50 μg/ml; kanamycin, 50 μg/ml; nalidixic acid, 25 μg/ml. *Streptomyces* strains were constructed by cross-genus conjugation with *E. coli* as previously described (35). Enzymes were purchased from New England BioLabs unless otherwise stated, and oligonucleotides were purchased from Integrated DNA Technologies, Inc. All of the strains, cosmids, and plasmids used in this study are described in Table S1 in the supplemental material, and all of the oligonucleotides and other synthetic DNAs used are provided in Table S2 in the supplemental material.

10.1128/mSphere.00305-16.1 Table S1  Bacterial strains, cosmids, fosmids, and plasmids used in this study. Download Table S1, DOCX file, 0.03 MB. Copyright © 2016 McLean et al. 2016 McLean et al. This content is distributed under the terms of the Creative Commons Attribution 4.0 International license.

10.1128/mSphere.00305-16.2 Table S2  Oligonucleotide primers and other synthetic DNAs used in this study. Download Table S2, DOCX file, 0.1 MB. Copyright © 2016 McLean et al. 2016 McLean et al. This content is distributed under the terms of the Creative Commons Attribution 4.0 International license.

10.1128/mSphere.00305-16.3 Table S3  FscRI^S4^ and putative orthologs encoded by antimycin producers. Download Table S3, DOCX file, 0.1 MB. Copyright © 2016 McLean et al. 2016 McLean et al. This content is distributed under the terms of the Creative Commons Attribution 4.0 International license.

### Construction of plasmids.

The insert for each plasmid generated in this study was prepared by PCR amplification with Q5 High-Fidelity DNA polymerase and oligonucleotides containing restriction sites. PCR-amplified inserts were restricted and cloned into the relevant plasmids cut with the same enzymes by standard molecular biology procedures. All clones were sequenced to verify the integrity of insert DNA. The restriction sites used for cloning are provided with the plasmid descriptions in Table S1 in the supplemental material.

### Design of the apramycin theophylline riboswitch cassette.

A λ Red recombineering template (pUC57-AprTheo) was designed and synthesized by MWG Biotech. The PCR template for recombineering was identical to that of pIJ773 (46), except that one end contained *ermE*p2 (39) repressed by a theophylline-controlled riboswitch (47). The synthesized cassette also contains an optional hexahistidine tag for knocking in a nickel affinity purification tag at the native locus. A schematic of the AprTheo PCR template is shown in Fig. S4 in the supplemental material, and further details concerning its design, including its DNA sequence, are available from FigShare at https://dx.doi.org/10.6084/m9.figshare.3838032.v1.

### Construction of pUC19-promKanprom.

To construct pUC19-promKanprom, the neomycin/kanamycin resistance marker from Supercos1 was PCR amplified with RFS444 and RFS445 and the product was used to replace the apramycin resistance gene and *oriT* of pIJ773 (46) by recombineering with *E. coli* GB05-red (48) to result in pIJ773KnFRT. Next, four PCR fragments were produced. (i) RFS406 and RFS407 were used to PCR amplify the kanamycin resistance from pIJ773KnFRT, (ii) RFS658 and RFS659 were used to PCR amplify the *rpsL*(XC) promoter from pCRISPomyces-2 (38, 49), (iii) RFS667 and RFS668 were used to PCR amplify the ermE* promoter from pSET152-*ermE*p, and (iv) RFS663 and RFS664 were used to linearize pUC19. The resulting PCR products were restricted with DpnI, gel purified, and assembled with the NEB HiFi DNA Assembly kit. The resulting plasmid, pUC19-promKanprom, contained a kanamycin resistance gene flanked by divergently firing *rpsL*(XC) and ermE* promoters. A schematic of the promKanprom PCR template is shown in Fig. S5 in the supplemental material, and its DNA sequence is available at http://www.ryanseipkelab.com/tools.html.

10.1128/mSphere.00305-16.8 Figure S5  Schematic of the promKanprom cassette. P1, prime site (TACGTCTCCGTCGTCTACTC) 1; P2, prime site (CATATGGGGCCTCCTGTTCT); *kanR*, kanamycin resistance; FRT sites, for excision of the resistance marker by the Flp recombinase. The sequence of the plasmid harboring this cassette is available at http://www.ryanseipkelab.com/tools.html. Download Figure S5, TIF file, 0.8 MB. Copyright © 2016 McLean et al. 2016 McLean et al. This content is distributed under the terms of the Creative Commons Attribution 4.0 International license.

### Cosmid manipulations.

The AprTheo PCR template described above was used to replace the *antB* or *antC* promoter harbored by cosmid 213 (12) by using RFS413 and RFS414 (*antB*p) or RFS415 and RFS416 (*antC*p) and the ReDirect PCR targeting system as previously described (46). The apramycin resistance gene and *oriT* were removed from modified cosmids with the FLP recombinase as previously described (46), resulting in cosmids 213-ABribo-FLP and 213-CDEribo-FLP. Cosmids 213-ABribo-FLP and 213-CDEribo-FLP were moved to *E. coli* GB05-red (48) and further engineered to harbor the ΦC31 integrase, *attP* site, and apramycin resistance gene originating from pIJ10702 (also known as pMJCOS1) (50) by RecET recombineering as previously described (48), resulting in cosmids 213-ABribo-FLP-ΦC31 and 213-CDEribo-FLP-ΦC31. Cosmid 213-ΦC31-BC-prom was constructed by replacing the *antB-antC* intragenic region of cosmid 213-ΦC31 by recombineering with GB05-red and a PCR product generated with pUC19-promKanprom and oligonucleotides RFS654 and RFS657. Thus, the *ant* gene cluster harbored by this final ΦC31 integrative construct is entirely controlled by divergently firing *rpsL*(XC) and ermE* promoters.

### Deletion of *fscRI*.

The *fscRI* gene was deleted by using the pCRISPomyces-2 system described previously (49). First, a single-guide RNA protospacer was generated by annealing oligonucleotides RFS574 and RFS575 and the resulting DNA fragment was cloned into the BbsI site of pCRISPomyces-2 by Golden Gate Assembly. Second, a homology-directed repair template consisting of ~3.8 kb of DNA homologous to the region adjacent to the Cas9-induced double-strand break was generated. The repair template was generated by sequentially cloning a HindIII-SpeI-restricted PCR fragment amplified with RFS521 and RFS522 into pIJ12738 (51) and then cloning a SpeI-KpnI-restricted PCR fragment generated with RFS523 and RFS524. The resulting plasmid (pIJ12738-*fscRI*-UPDN) was used as a PCR template with RFS572 and RFS573, and the resulting PCR product was restricted with XbaI and cloned into pCRISPomyces-2 containing the protospacer targeting *fscRI*. The resulting CRISPR/Cas9 editing plasmid, pCRISPomyces-2-*fscRI*, was mobilized to S4 by conjugal transfer from *E. coli* ET12567/pUZ8002 as previously described (35). Temperature-sensitive pCRISPomyces-2-*fscRI* was cured from a single apramycin-resistant transconjugant by passage in LB at 37°C (two rounds) prior to the cultivation of a sporulated lawn on MS agar at 37°C. The resulting spores were serially diluted, and 11 single colonies were replica plated to assess apramycin sensitivity. Five apramycin-sensitive colonies were obtained and subsequently evaluated for the absence of *fscRI* by polymorphic shift PCR with RFS598 and RFS599. The integrity of the resulting Δ*fscRI* null mutant was verified by DNA sequencing.

### Chemical analysis.

*Streptomyces* strains were cultured in MS broth (50 ml in a 250-ml flask) while shaking (180 rpm) at 27°C for 7 days. MS broth was supplemented with theophylline (4 mM) from the onset of culturing as required for M1146 strains. Bacterial cells were removed by centrifugation, and metabolites were extracted from the supernatant with a Phenomenex strata-XL C_18_ (100 μm, 30 mg, 1 ml) solid-phase extraction (SPE) column and a vacuum manifold. The column was first washed with 1 ml of 100% methanol followed by 1 ml of deionized water. The column was then loaded with supernatant (10 ml in total) prior to being washed with 1 ml of deionized water and then washed with 2 ml of 30% methanol. Metabolites were eluted from the SPE column in 100% methanol (0.3 ml). Equal amounts of methanolic extract from two independent replicates of each strain were mixed and centrifuged for 10 min at 16,000 × *g* just prior to injection in order to remove insoluble material. Only the supernatant (2 μl) was injected into a Bruker MaXis Impact time of flight mass spectrometer and equipped with a Dionex UltiMate 3000 high-performance liquid chromatography apparatus with the same parameters as described previously (15).

### Bioassays.

Bioassays were performed essentially as described previously (52), except that 7 instead of 5 ml of soft nutrient agar was used and *Streptomyces* strains were cultivated at 30°C for 7 (instead of 10) days prior to a challenge with *C. albicans* CA6 (53). MS agar was supplemented with 2 mM theophylline for M1146 strains as required. Photographs of bioassay plates were taken ~48 h after the challenge.

### MIC and FIC index determination.

MIC assays were performed with 96-well flat-bottom microtitration plates in accordance with the Clinical and Laboratory Standards Institute guidelines adapted for *C. albicans* (54). The FIC index was determined according to reference 55 by evaluating the growth of *C. albicans* exposed to increasing pairwise concentrations of antimycin (0.0625 to 4 µg/ml) and candicidin (1 to 64 µg/ml). The formula used was FIC index = FIC A + FIC B, where FIC A = MIC of combination/MIC of compound A and FIC B = MIC of combination/MIC of compound B.

### Protein expression.

Recombinant His_6_-FscRI and FscRI-His_6_ were produced with *E. coli* Rosetta BL21(DE3) by using pET28a-*fscRI* and pET30a-*fscRI*, respectively. Production of His_6_-FscRI and FscRI-His_6_ was induced in mid-log phase by the addition of 1.5 mM isopropyl-β-d-thiogalactopyranoside. After 4 h of induction at 28°C, cells were harvested by centrifugation. The cell pellet was resuspended in 1× BugBuster Protein Extraction Reagent (Novagen) diluted with 50 mM Tris-Cl (pH 8.0), 200 mM NaCl, 20 mM imidazole, and 2 U of DNase I and incubated room temperature for 20 min. Insoluble material was removed from the lysate by centrifugation (16,000 × *g* for 10 min). Soluble and insoluble fractions were visualized with InstantBlue (Expedeon) after 15% SDS-PAGE.

### ChIP-sequencing and bioinformatic analyses.

Wild-type or Δ*fscRI*/pSETNFLAG-*fscRI* mutant S4 bacteria were cultivated for 2 days in LB while shaking at 200 rpm at 28°C and processed for ChIP-sequencing exactly as described previously, with anti-FLAG M2 agarose beads (Sigma) (56), except that an Active Motif, Inc., EpiShear sonicator (30% amplitude, 30 s on and 30 s off for a total time of 13 min) was used to shear DNA to an average size of approximately 200 to 300 nucleotides. The pure DNA resulting from immunoprecipitates from two biological replicates of wild-type and Δ*fscRI*/pSETNFLAG-*fscRI* mutant S4 bacteria, as well as nonimmunoprecipitated chromosomal DNA, were sequenced with the Illumina HiSeq3000 platform with 150-nucleotide paired-end reads by the University of Leeds Next Generation Sequencing Facility at the St. James Teaching Hospital NHS Trust. The forward reads were mapped to the S4 genome with Bowtie 2 version 2.1.0 (57), and the resulting alignments were converted from the .SAM to the .BAM format and sorted according to chromosomal position with SAMtools version 1.1 (58). The aligned and sorted .BAM files for immunoprecipitated samples were converted to the bigWig format and normalized by read count compared to the DNA-only input control by using the default settings of deepTools bamCompare version 2.3.3 (59), with the exception that flag --ratio=subtract was used instead of the default --ratio=log2. This resulted in a single bigWig file for each treatment, which was visualized with Integrated Genomics Viewer version 2.3.78 (60). Plots were generated with the deepTools programs computeMatrix and plotProfile (59) with a bin size of 50 and a custom .BED file specifying the region displayed.

### Accession number(s).

The next-generation sequencing data obtained in this study are available under ArrayExpress accession no. E-MTAB-5122. The DNA sequences of tools constructed during this study are available at http://www.ryanseipkelab.com/tools.html.
