# Supplementary figures and images for: Coordinate Regulation of Antimycin and Candicidin Biosynthesis
Source: mSphere. 2016 Dec 7;1(6):e00305-16. doi: 10.1128/mSphere.00305-16 (PMC5143413; doi:10.1128/mSphere.00305-16)

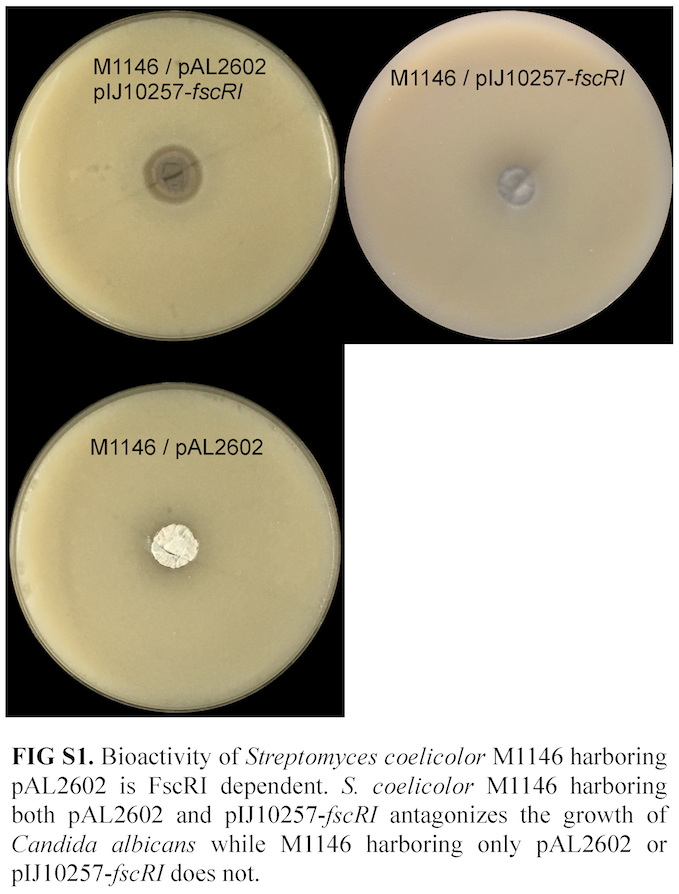

Supplement: Figure S1 [file sph006162205sf4.tif]

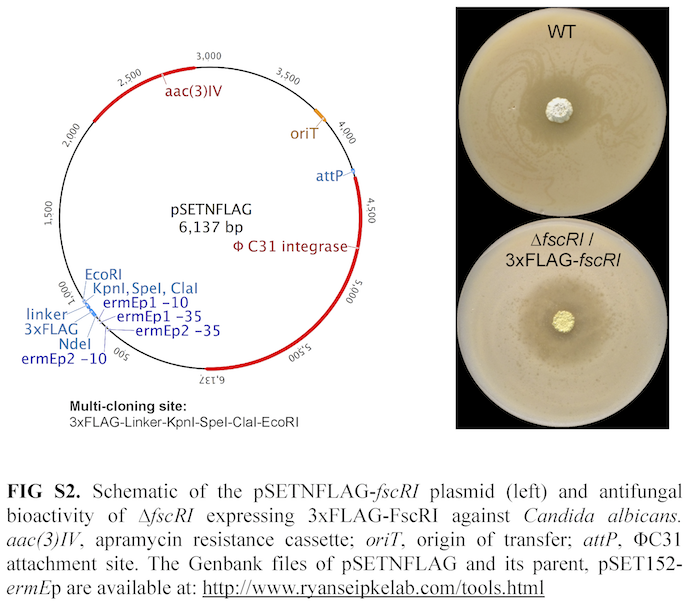

Supplement: Figure S2 [file sph006162205sf5.tif]

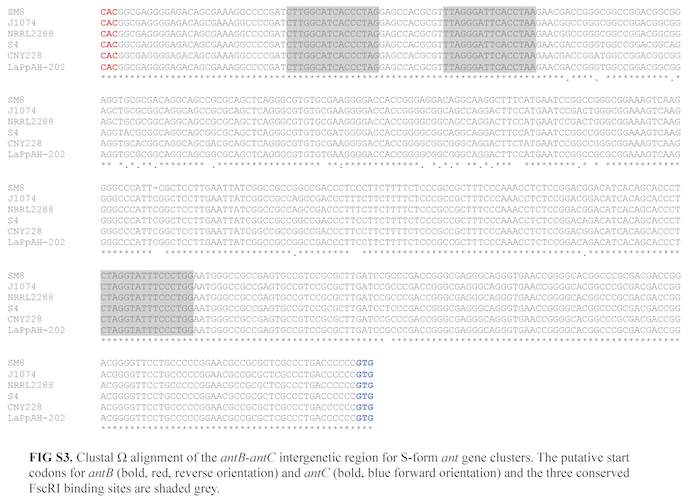

Supplement: Figure S3 [file sph006162205sf6.tif]

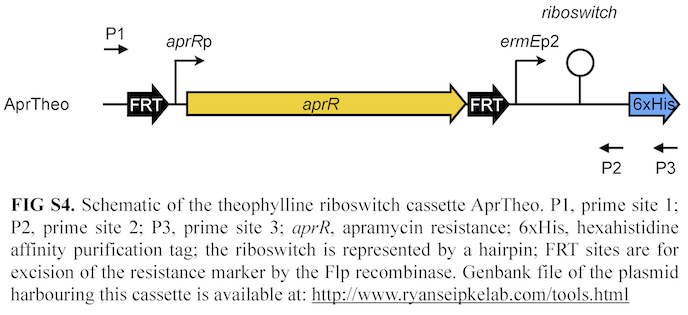

Supplement: Figure S4 [file sph006162205sf7.tif]

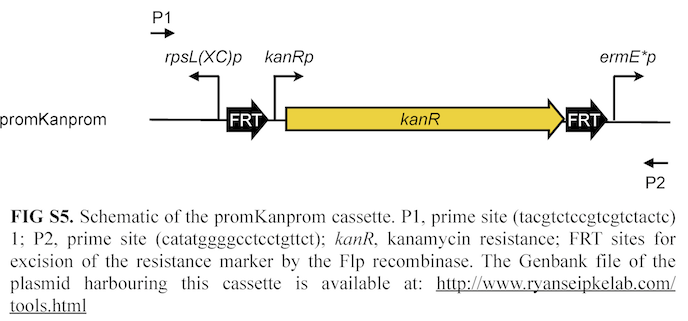

Supplement: Figure S5 [file sph006162205sf8.tif]
