# Supplementary material for: Coordinate Regulation of Antimycin and Candicidin Biosynthesis
Source: mSphere. 2016 Dec 7;1(6):e00305-16. doi: 10.1128/mSphere.00305-16 (PMC5143413; doi:10.1128/mSphere.00305-16)
Supplement: Table S1 [file sph006162205st1.docx]

**Table S1.** Bacterial strains, cosmids, fosmids and plasmids used in this study

| **Strain/cosmid/plasmid** | **Description ^a^** | **Reference** |
| --- | --- | --- |
| ***Streptomyces*** |  |  |
| S4 | Wild type *Streptomyces albus* S4 | (1) |
| ∆*fscRI* | S4 *fscRI* null mutant | This study |
| ∆*fscRI attB* ΦBT1::pIJ10257 | *fscRI* null mutant harboring the empty pIJ10257 vector; Hyg^R^ | This study |
| ∆*fscRI attB* ΦBT1::pIJ10257-*fscRI* | *fscRI* null mutant complemented with *fscRI* expressed from the *ermE** promoter; Hyg^R^ | This study |
| ∆*fscRI attB* ΦC31::pSETNFLAG | *fscRI* null mutant harboring the empty pSETNFLAG vector; Apr^R^ | This study |
| ∆*fscRI attB* ΦC31::pSETNFLAG-*fscRI* | *fscRI* null mutant complemented with 3xFLAG-*fscRI* expressed from the *ermE** promoter; Apr^R^ | This study |
| M1146 | *S. coelicolor* M145 harboring mutations in the biosynthetic pathways for: actinorhodin, undecylprodigiosin, calcium-dependent antibiotic and coelimycin | (2) |
| M1146 *attB* ΦC31::Cosmid213 | M1146 derivative harboring Cosmid 213; Carb^R^, Apr^R^ | This study |
| M1146 *attB* ΦC31::Cosmid213-BC-prom | M1146 derivative harboring Cosmid 213-BC-prom; Carb^R^, Kan^R^, Apr^R^ | This study |
| M1146 *attB* ΦC31::Cosmid213-ABribo-FLP | M1146 derivative harboring Cosmid 213-ABribo-FLP; Carb^R^, Apr^R^ | This study |
| M1146 *attB* ΦC31::Cosmid213-CDEribo-FLP | M1146 derivative harboring Cosmid 213-CDEribo-FLP; Carb^R^, Apr^R^ | This study |
| M1146 *attB* ΦC31::Cosmid213 *attB* ΦBT1::pIJ10257 | M1146 derivative harboring Cosmid 213 and pIJ10257; Carb^R^, Apr^R^, Hyg^R^ | This study |
| M1146 *attB* ΦC31::Cosmid213 *attB* ΦBT1::pIJ10257-*fscRI* | M1146 derivative harboring Cosmid 213 and pIJ10257-*fscRI*; Carb^R^, Apr^R^, Hyg^R^ | This study |
| M1146 *attB* ΦC31::Cosmid213-ABribo-FLP *attB* ΦBT1::pIJ10257-*fscRI* | M1146 derivative harboring Cosmid 213-ABribo-FLP and pIJ10257-*fscRI*; Carb^R^, Apr^R^, Hyg^R^ | This study |
| M1146 *attB* ΦC31::Cosmid213-CDEribo-FLP ΦBT1::pIJ10257-*fscRI* | M1146 derivative harboring Cosmid 213-CDEribo-FLP and pIJ10257-*fscRI*; Carb^R^, Apr^R^, Hyg^R^ | This study |
| M1146 *attB* ΦC31::Cosmid213-ABCDEribo-FLP | M1146 derivative harboring Cosmid 213-CDEribo-FLP and pIJ10257-*fscRI*; Carb^R^, Apr^R^, Hyg^R^ | This study |
| ***Escherichia coli*** |  |  |
| BL21 | Host for heterologous protein expression | Novagen |
| BW25113 | Host for REDIRECT PCR targeting system | (3) |
| TOP10 | General cloning host | Invitrogen |
| ET12567 | Non-methylating host for transfer of DNA into *Streptomyces* spp. (*dam, dcm, hsdM*); Cam^R^ | (4) |
| XL1-Blue | General cloning host | Agilent Technologies |
| GB0R-red | Host for RecET recombination | (5) |
| **Cosmids and fosmids** |  |  |
| Supercos1 | Cosmid backbone for *S. albus* S4 Cosmid 213; Carb^R^, Kan^R^ | Stratagene |
| Cosmid 213 | Supercos1 derivative containing the entire antimycin gene cluster; Carb^R^, Kan^R^ | This study |
| Cosmid 213-ABribo-FLP | Cosmid 213 derivative with *antBA* expression controlled by a theophylline inducible riboswitch; Carb^R^, Kan^R^ | This study |
| Cosmid 213-CDEribo-FLP | Cosmid 213 derivative with *antCDE* expression controlled by a theophylline inducible riboswitch; Carb^R^, Kan^R^ | This study |
| Cosmid 213-ΦC31 | Cosmid 213 derivative engineered to integrate into the ΦC31 *attB* site; Carb^R^, Apr^R^ | This study |
| Cosmid 213-ΦC31-BC-prom | Cosmid 213-ΦC31derivative with *antBC* and *antCDE* expression controlled by *rpsL*(XC) and *ermE** promoters, respectively; Carb^R^, Kan^R^, Apr^R^ | This study |
| Cosmid 213-ABribo-FLP- ΦC31 | Cosmid 213-ABribo-FLP derivative engineered to integrate into the ΦC31 *attB* site; Carb^R^, Apr^R^ | This study |
| Cosmid 213-CDEribo-FLP- ΦC31 | Cosmid 213-CDEribo-FLP derivative engineered to integrate into the ΦC31 *attB* site; Carb^R^, Apr^R^ | This study |
| pAL2602 | ΦC31 integrative fosmid clone harboring the antimycin gene cluster from *Streptomyces* sp. NRRL 2288; Apr^R^ | (6) |
| **Plasmids** |  |  |
| pCRISPomyces-2 | pGM1190 derivative harboring the CRISPR/Cas9 machinery; Apr^R^ | (7) |
| pCRISPomyces-2-*fscRI* | Derivative of pCRISPomyces-2 derivative containing the *fscRI* targeting protospacer cloned into the BbsI site and homology-directed repair arms cloned into the XbaI site; Apr^R^ | This study |
| pET28a | Commercial protein expression vector; Kan^R^ | Novagen |
| pET30a | Commercial protein expression vector; Kan^R^ | Novagen |
| pET28a-*fscRI* | pET28a derivative containing *fscRI* cloned into the NdeI-HindIII sites; Kan^R^ | This study |
| pET30a-*fscRI* | pET30a derivative containing the *fscRI* without a stop codon cloned into the NdeI-HindIII sites; Kan^R^ | This study |
| pSET152 | *E. coli* – *Streptomyces* shuttle vector, integrates into the ΦC31 *attB* site in actinomycetes; Apr^R^ | (8) |
| pSET152*ermE*p | pSET152 derivative containing *ermE**p cloned into the EcoRV-EcoRI sites; Apr^R^ | This study |
| pSETNFLAG | pSET152*ermE** derivative with an N-terminal 3xFLAG tag and multi-cloning site cloned into the NdeI-KpnI sites; Apr^R^ | This study |
| pSETNFLAG-*fscRI* | pSETNFLAG derivative harboring *fscRI* cloned into KpnI-EcoRI sites; Apr^R^ | This study |
| pIJ773 | ReDirect PCR template plasmid harboring an apramycin resistance cassette; Carb^R^, Apr^R^ | (3) |
| pIJ773KnFRT | pIJ773 derivative where the apramycin resistance gene has been replaced by the neomycin/kanamycin resistance gene from Supecos1; Carb^R^, Kan^R^ | This study |
| pIJ10257 | pMS81 derivative containing *ermE**p, integrates into the ΦBT1 *attB* site in *Streptomyces*; Hyg^R^ | (9) |
| pIJ10257-*fscRI* | pIJ10257 derivative containing the *fscRI* coding sequence cloned into the NdeI-HindIII sites | This study |
| pIJ10702 | Supercos1 derivative harboring the ΦC31 integrase, *attP*, *oriT* and apramycin resistance gene from pSET152; Carb^R^, Apr^R^ | (10) |
| pIJ12738 | Conjugative vector, non-replicative in *Streptomyces*; Apr^R^ | (11) |
| pIJ12738-*fscRI-*UPDN | pIJ12738 derivative harboring the downstream *fscRI* homology-directed repair arms cloned into the KpnI-HindIII sites; Apr^R^ | This study |
| pUC19 | General cloning plasmid; Carb^R^ | New England Biolabs |
| pUC19-promKanprom | pUC19 derivative harboring a kanamycin resistance gene flanked by divergently firing *rpsL*(XC) and *ermE** promoters; Carb^R^, Kan^R^ | This study |
| pUC57-AprTheo | pUC57 derivative harboring the commercially synthesised apramycin theophylline riboswitch cassette; Carb^R^, Apr^R^ | This study |
| pUZ8002 | Encodes conjugation machinery for mobilization of plasmids from *E. coli* to *Streptomyces;* Kan^R^ | (4) |

^a^ Carb, carbenicillin; Apr, apramycin; Hyg, hygromycin, Kan, kanamycin; Cam, chloramphenicol; *oriT*, origin of conjugal transfer

**References:**

1. **Barke J**, **Seipke RF**, **Grüschow S**, **Heavens D**, **Drou N**, **Bibb MJ**, **Goss RJM**, **Yu DW**, **Hutchings MI**. 2010. A mixed community of actinomycetes produce multiple antibiotics for the fungus farming ant *Acromyrmex octospinosus*. BMC Biol **8**:109.

2. **Gomez-Escribano JP**, **Bibb MJ**. 2011. Engineering *Streptomyces coelicolor* for heterologous expression of secondary metabolite gene clusters. Microbial Biotechnology **4**:207–215.

3. **Gust B**, **Challis GL**, **Fowler K**, **Kieser T**, **Chater KF**. 2003. PCR-targeted *Streptomyces* gene replacement identifies a protein domain needed for biosynthesis of the sesquiterpene soil odor geosmin. Proc Natl Acad Sci USA **100**:1541–1546.

4. **MacNeil DJ**, **Gewain KM**, **Ruby CL**, **Dezeny G**, **Gibbons PH**, **MacNeil T**. 1992. Analysis of Streptomyces avermitilis genes required for avermectin biosynthesis utilizing a novel integration vector. Gene **111**:61–68.

5. **Fu J**, **Bian X**, **Hu S**, **Wang H**, **Huang F**, **Seibert PM**, **Plaza A**, **Xia L**, **Müller R**, **Stewart AF**, **Zhang Y**. 2012. Full-length RecE enhances linear-linear homologous recombination and facilitates direct cloning for bioprospecting. Nat Biotechnol **30**:440–446.

6. **Yan Y**, **Zhang L**, **Ito T**, **Qu X**, **Asakawa Y**, **Awakawa T**, **Abe I**, **Liu W**. 2012. Biosynthetic pathway for high structural diversity of a common dilactone core in antimycin production. Org Lett **14**:4142–4145.

7. **Cobb RE**, **Wang Y, Zhao H**. 2015. High-Efficiency multiplex genome editing of *Streptomyces* species using an engineered CRISPR/Cas System. ACS Synth Biol **14:**723–728.

8. **Kieser TB**, **Buttner MJ**, **Chater MJ**, **Hopwood KF**. 2000. Practical *Streptomyces* genetics. The John Innes Foundation, Norwich, United Kingdom.

9. **Hong H-J**, **Hutchings MI**, **Hill LM**, **Buttner MJ**. 2005. The role of the novel Fem protein VanK in vancomycin resistance in *Streptomyces coelicolor*. J Biol Chem **280:**13055–13061.

10. **Yanai K**, **Murakami T**, **Bibb M**. 2006. Amplification of the entire kanamycin biosynthetic gene cluster during empirical strain improvement of *Streptomyces kanamyceticus.* Proc Natl Acad Sci USA **103:**9661–9666.

11. **Fernández-Martínez LT**, **Bibb MJ**. 2014. Use of the Meganuclease I-SceI of *Saccharomyces cerevisiae* to select for gene deletions in actinomycetes. Sci Rep **4:**7100.
