# Supplementary material for: Coordinate Regulation of Antimycin and Candicidin Biosynthesis
Source: mSphere. 2016 Dec 7;1(6):e00305-16. doi: 10.1128/mSphere.00305-16 (PMC5143413; doi:10.1128/mSphere.00305-16)
Supplement: Table S2 [file sph006162205st2.docx]

**Table S2.** Oligonucleotide primers and other synthetic DNAs used in this study

| **Primer alias** | **Sequence (5'-3')*** | **Description** |
| --- | --- | --- |
| RFS406 | tgtaggctggagctgcttc | PCR: KnFRT cassette |
| RFS407 | attccggggatccgtcgac | PCR: KnFRT cassette |
| RFS413 | tagagggtgctgtgatgtctgtccggagaggtttgggaatgtaggctggagctgcttc | PCR: *antB* promoter riboswitch recombineering |
| RFS414 | ggttctccacattgagtgcggagcccctgtcacgcctcatcttgttgccccttctcagg | PCR: *antB* promoter riboswitch recombineering |
| RFS415 | ttcccaaacctctccggacagacatcacagcaccctctatgtaggctggagctgcttc | PCR: *antC* promoter riboswitch recombineering |
| RFS416 | acatgacaccaaccctcggttgcgagcaggtacttctcatcttgttgccccttctcagg | PCR: *antC* promoter riboswitch recombineering |
| RFS424 | ata**catatg**gatcccgcgccggcggc | PCR: *fscRI* coding sequence |
| RFS425 | tat**aagctt**tcacttgatgaagtcct | PCR: *fscRI* coding sequence |
| RFS444 | ggaataggaacttatgagctcagccaatcgactggcgagcgaagccctgcaaagtaaact | PCR: KnFRT recombineering template |
| RFS445 | cagttcgaagttcctattctctagaaagtataggaacttctcagaagaactcgtcaagaa | PCR: KnFRT recombineering cassette |
| RFS521 | ata**aagctt**gccctggtccacatcgag | PCR: *fscRI* homology-directed repair arm |
| RFS522 | ata**actagt**gatccatgagcgtgctgtg | PCR: *fscRI* homology-directed repair arm |
| RFS523 | ataactagtcctcaaggtcggcacctg | PCR: *fscRI* homology-directed repair arm |
| RFS524 | ataggtaccggtggcgtcctggagtg | PCR: *fscRI* homology-directed repair arm |
| RFS572 | ataata**tctaga**gccctggtccacatcgag | PCR: *fscRI* homology-directed repair arm |
| RFS573 | tatata**tctaga**ggtggcgtcctggagtg | PCR: *fscRI* homology-directed repair arm |
| RFS574 | aggccggaggacgagccgccgaag | CRISPR protospacer targeting *fscRI* |
| RFS575 | aaaccttcggcggctcgtcctccg | CRISPR protospacer targeting *fscRI* |
| RFS582 | atatat**gatatc**agcccgacccgagcacg | PCR: construction of pSET152*ermE*p |
| RFS583 | tatata**gaattcatcgatactagtggtacc**atgcaggactctagtta | PCR: construction of pSET152*ermE*p |
| RFS594 | tatata**catatg**gactacaa | PCR: construction of pSET152NFLAG |
| RFS595 | atatat**ggtacc**actaccgc | PCR: construction of pSET152NFLAG |
| RFS598 | gggctaccacagtattg | PCR: confirmation of ∆*fscRI* mutant strain |
| RFS599 | gtcgaagacggtgactc | PCR: confirmation of ∆*fscRI* mutant strain |
| RFS600 | tatata**aagctt**cttgatgaagtcctcga | PCR: *fscRI* coding sequence without stop codon |
| RFS601 | tatata**atcgat**cttgatgaagtcctcga | PCR: *fscRI* coding sequence without stop codon |
| RFS602 | atatat**ggtacc**gatcccgcgccggcggc | PCR: *fscRI* coding sequence |
| RFS603 | tatata**gaattc**tcacttgatgaagtcct | PCR: *fscRI* coding sequence |
| RFS654 | tctccacattgagtgcggagcccctgtcacgcctcactacgtctccgtcgtctactc | PCR: *antB-antC* *rpsL*(XC)-Kan-*ermE** recombineering |
| RFS657 | cagacatgacaccaaccctcggttgcgagcaggtacttctcatatggggcctcctgttct | PCR: *antB-antC* *rpsL*(XC)-Kan-*ermE** recombineering |
| RFS663 | actggccgtcgttttacaac | PCR: pUC19 |
| RFS664 | gaattcgagctcggtacccg | PCR: pUC19 |
| RFS665 | gttgtaaaacgacggccagtcattacgtctccgtcgtcta | PCR: *rpsL*(XC) promoter |
| RFS666 | gaagcagctccagcctacagccctgcaggcggaagtcag | PCR: *rpsL*(XC) promoter |
| RFS667 | ggtcgacggatccccggaatagcccgacccgagcacgcgc | PCR: *ermE** promoter |
| RFS668 | cgggtaccgagctcgaattccatatggggcctcctgttct | PCR: *ermE** promoter |
| NFLAG Gblock | tatata**catatg**gactacaaggaccacgacggcgactacaaggaccacgacatcgactacaaggacgatgacgacaagggtggaggcggttcaggcggaggtggctctggcggtggcggtagt**ggtacc**atatat | Gblock for construction of pSETNFLAG |

* non-homologous sequences are underlined and engineered restriction endonuclease sites are bolded
