# Supplementary material for: Coordinate Regulation of Antimycin and Candicidin Biosynthesis
Source: mSphere. 2016 Dec 7;1(6):e00305-16. doi: 10.1128/mSphere.00305-16 (PMC5143413; doi:10.1128/mSphere.00305-16)
Supplement: Table S3 [file sph006162205st3.docx]

**Table S3.** FscRI^S4^ and putative orthologs encoded by antimycin producers

| ***Streptomyces* species** | **Genome accession number** | **Class of *ant* gene cluster** | **Amino acid sequence** | **Shared amino acid identity (%)** |
| --- | --- | --- | --- | --- |
| *S. albus* S4 | CADY00000000.1 | S-form | MDPAPAADPAGSPERAAVLADGFDRAGAYVACLDPSLTIQQVNQEFERRFGGSSSELCGSRFCDLVHPSVQQPLMHQFARMLDGKRHRFATEVIAVDQERTASTLPLNALAVRGGRTPDVAAILVVMNAAEEEAGDADVMAPRKKLLSEIDARILEGIAAGVSTIPLASRLYLSRQGVEYHVTGLLRKLKVPNRAALVSRAYSMGVLKVGTWPPKVVEDFIK | 100 |
| *S. albus* J1074 | NC_020990 | S-form | MDPAPAADPAGSPERAAVLADGFDRAGAYVACLDPSLTIQQVNQEFERRFGGSSSELCGSRFCDLVHPSVQQPLMHQFARMLDGKRHRFATEVIAVDQERTASTLPLNALAVRGGRTPDVAAILVVMNAAEEEAGDADVMAPRKKLLSEIDARILEGIAAGVSTIPLASRLYLSRQGVEYHVTGLLRKLKVPNRAALVSRAYSMGVLKVGTWPPKVVEDFIK | 100 |
| *S. sp.* CNY228 | ARIN00000000.1 | S-form | MDPAPAADPAGSPERAAVLADGFDRAGAYVACLDPSLTIQQVNQEFERRFGGSSSELCGSRFCDLVHPSVQQPLMHQFARMLDGKRHRFATEVIAVDQERTASTLPLNALAVRGGRTPDVAAILVVMNAAEEEAGDADVMAPRKKLLSEIDARILEGIAAGVSTIPLASRLYLSRQGVEYHVTGLLRKLKVPNRAALVSRAYSMGVLKVGTWPPKVVEDFIK | 100 |
| S. sp. SM8 | AMPN00000000.1 | S-form | MDPAPAADPAGSPERAAVLADGFDRAGAYVACLDPSLTIQQVNQEFERRFGGSSSELCGSRFCDLVHPSVQQPLMHQFARMLDGKRHRFATEVIAVDQERTASTLPLNALAVRGGRTPDVAAILVVMNAAEEEAGDADVMAPRKKLLSEIDARILEGIAAGVSTIPLASRLYLSRQGVEYHVTGLLRKLKVPNRAALVSRAYSMGVLKVGTWPPKVVEDFIK | 100 |
| *S. sp.* LaPpAH-202 | ARDM00000000.1 | S-form | MDPAPAADPAGSPERAAVLADGFDRAGAYVACLDPSLTIQQVNQEFERRFGGSSSELCGSRFCDLVHPSVQQPLMHQFARMLDGKRHRFATEVIAVDQERTASTLPLNALAVRGGRTPNVAAILVVMNAAEEEAGDADVMAPRKKLLSEIDARILEGIAAGVSTIPLASRLYLSRQGVEYHVTGLLRKLKVPNRAALVSRAYSMGVLKVGTWPPKVVEDFIK | 99 |
| *Streptomyces* sp. TOR3209 | ARTR00000000.1 | I-form | MDRTPVAGPAGTAVPAAGHTDGFDRADAYIACLDPALTIQQVNQEFDRRFGGPASSLCGRNFCDLIHPSVRPPLMQQFSRLLEGKRRRFLTDVIAVDQESTASALPLRAMAVQGGHTPDVAAILVVMSGADERTEDAEEMAPRKKLLSEIDARILEGIAAGVSTIPLASRLFLSRQGVEYHVTGLLRALKVPNRAALVSRAYSMGVLKVGTWPPKVVEDYIK | 79 |
| *S. gancidicus* BKS 13-15 | AOHP00000000.1 | L-form | VTGAPHNRDRRSPSLHAAAHRNAPESRTPAPGNRRFYTAHIDPDIQIVAAEPDFSRQFGRTSADTCGRSLYELLHPSAPSVLNRHFTRLSEGRSARFAERMVGLGNAGRVFSGELTGIAVQNTTGRLAGIVVQVRPDTEADTTDGKDVIGPPRERLLSKLDAQVLEGIAAGASTVQLAARLYLSRQGVEYHVGLMLRKLKAPNRAALVARAHSMGMLTVGQWPPRVLPEFIK | 46 |
| ,*S. hygroscopicus* subsp. *jinggangensis* 5008 | NC_017765 | L-form | VAAEHRTTERFSDICWSVFVQSGFCIAHLDPRLRISAANGPFCSHIGSSPADVLGRDILDYLHPGVREKVRREFARLADGRSARFADDVIVVDAEGKSFQAELTGVAVHGSASARVEGIVVLLRPSGSRSPGVAPARQKLFSPVHARVLEGVAAGESTVQLASRLFLSRGGVEYHVASLLRKMKVANRPALISKGYALGVFAVGEWPPRVQPEFIAS | 44 |
| *S. hygroscopicus* subsp. *jinggangensis* TL01 | NC_020895 | L-form | VAAEHRTTERFSDICWSVFVQSGFCIAHLDPRLRISAANGPFCSHIGSSPADVLGRDILDYLHPGVREKVRREFARLADGRSARFADDVIVVDAEGKSFQAELTGVAVHGSASARVEGIVVLLRPSGSRSPGVAPARQKLFSPVHARVLEGVAAGESTVQLASRLFLSRGGVEYHVASLLRKMKVANRPALISKGYALGVFAVGEWPPRVQPEFIAS | 44 |
| *Streptomyces* sp. 303MFCol5.2 | ARTR00000000.1 | I-form | MSVVTTSITASSTTVVKAGAGVNRRRTYTAHVCPKGMTITAAEADFAAQFGASPGQICDRTLSDLLRAGTPEVLRHRFTDLSEGRTSWFTERVAGRHDSGRVFAADLTGIAVTGATGPAGLVLLLSPLGAAGEPYPRELTLSELDVQVLEGVAGGASTVQLAGRLYLSRQGVEYRVRLLLRRFDAPNRPALVARAHALGLFAPGQWPPRVLPELIE | 37 |
| *S. ambofaciens* ATCC 23877 | AM238663 | L-form | VATTSFSDASPGQQRNAAAPAAPAHRVPTGGGAHRGAASADAWTAHVSPGDPVVTAAEPEFARQFGLSADEIRGRRLLDLLRSPVPARLREQFTFLSSGRCRRFTETVTYRDGTGRDFPAELTGVAVRKPSGDVFGVVILLRRAGAAHRAAEMRRAGDRRPPQKGTLAEAAGRPVLSALDAQVLEGVARGESTAQLASRLYLSRQGIEYRVGQMLRRFEAPNRPALVARAHALGMFAPGQWPPRVLPERVK | 36 |
| *S. griseoflavus* Tü4000 | ACFA00000000.1 | L-form | VATKSYPDASPSKKRTAATAVPARRHLITAQDHVTPAATCTAHLSPQDLVVTAAEPEFARQFGLSADEICGRGLLELLRSRTPGHLREQFAALSSGPGRRFKQKVTGRDGDGRSFHADITAIAVRQPSGEMAGVVVLLRRTAEAVTGGPVLSALDAQVLEGVASGESTVQLASRLYLSRQGIEYRVGQMLRRFDAPNRPALVARAHALGMFAAGQWPPRVLPECVR | 36 |
